# Optimization of the Encapsulation of Lemuru Fish Protein Hydrolysate by Spray-Drying Using Response Surface Methodology

**DOI:** 10.17113/ftb.63.01.25.8626

**Published:** 2025-03

**Authors:** Ayu Hanifah, Wawan Kosasih, Diah Ratnaningrum, Dian Andriani, Herlian Eriska Putra, Yellianty Yelliantty, Sri Priatni

**Affiliations:** 1 Research Center for Applied Microbiology, National Research and Innovation Agency Republic of Indonesia, Jl. Raya Jakarta-Bogor Km, 46, Cibinong, Bogor, Jawa Barat 16911, Indonesia; 2 Directorate of Laboratory Management, Research Facilities and Science and Technology areas, KST Samaun Samadikun, Gd Basics Tw 1, Lt 1, Cisitu-Sangkuriang, Bandung 40135, Indonesia; 3 Research Center for Environment and Clean Technology, National Research and Innovation Agency Republic of Indonesia, KST BJ Habiebie Gd. 720 Setu Tangerang Selatan Banten 15314, Indonesia; 4 Department of Food Technology, Faculty of Engineering, Universitas Pasundan Jalan Setiabudi 193 Bandung, West Java 40135, Indonesia

**Keywords:** encapsulation, protein hydrolysate, lemuru fish, spraydrying, RSM

## Abstract

**Research background:**

Encapsulating lemuru fish protein hydrolysate is important to maintain its stability. However, optimal conditions for the encapsulation process of lemuru fish protein hydrolysate using statistical methods remain unexplored. This study aims to address this problem by optimizing the encapsulation conditions.

**Experimental approach:**

Maltodextrin and gum Arabic were used as carrier agents, with mass per volume ratio ranging from 10 to 30 %, and spray dryer inlet temperatures between 90 and 100 °C. In this study, we analysed the main interactions of these variables using response surface methodology (RSM).

**Results and conclusions:**

Our results show that mass per volume ratio of maltodextrin of 25 % and inlet temperature of 100 °C are the optimal conditions for the encapsulation of fish protein hydrolysate. The optimal conditions resulted in a high desirability index of 0.864, indicating an effective balance between yield, solubility and hygroscopicity. The actual results also fall well within the confidence interval of the predicted values, confirming the robustness of the model and the reliability of the predicted optimal encapsulation conditions. Encapsulated fish protein hydrolysate was compared with its non-encapsulated counterpart and characterised using Fourier transform infrared spectroscopy (FTIR), scanning electron microscopy (SEM), and particle size analyser (PSA) to validate the results. The encapsulated fish protein hydrolysate showed distinct properties, such as the presence of functional groups from maltodextrin, interconnected particle and more homogeneous and narrower particle size distribution.

**Novelty and scientific contribution:**

The encapsulation of lemuru fish protein hydrolysate using maltodextrin with mass per volume ratio of 25 % and inlet temperature 100 °C was successful in improving the properties of the protein hydrolysate. Further research should explore the functional properties of fish protein hydrolysate.

## INTRODUCTION

The significant health benefits are the reason for the growing global interest in functional foods and nutraceuticals. These foods, enriched with bioactive compounds, play a crucial role in improving nutrition, preventing disease and promoting general health. A key contributor to the development of functional foods is the fishing industry, especially in countries like Indonesia, where fishery productivity has increased significantly, from less than 7 million tonnes in previous years to over 7.2 million tonnes in 2021 (*1*). Among the diverse range of seafood, the lemuru fish, a small pelagic fish species, stands out due to its significant contribution to the Indonesian marine market. The nutritional profile of lemuru fish is outstanding. It is rich in high-quality protein, essential amino acids, vitamins and nutrients, making it an excellent candidate for the formulation of fish protein hydrolysate (*2*, *3*).

Fish protein hydrolysate is a mixture of low-molecular-mass peptides with 2–20 amino acids produced by acid, alkaline or enzymatic hydrolysis. Among these methods, enzymatic hydrolysis is the most promising to produce fish protein hydrolysate that is highly functional and nutritious. Fish protein hydrolysate has the potential to be used in the development of nutraceuticals and pharmaceuticals, as it is effective in treating cardiovascular disease, cancer and inflammation (*4*). Furthermore, fish protein hydrolysate has different biological activities, such as antioxidant, antihypertensive and anti-obesity, making it a good alternative for functional food production (*5*). Due to these properties, there is a growing interest in it in the food and nutraceutical industry (*6*). Despite these benefits, fish protein hydrolysates are unstable due to their high protein content. The instability leads to several challenges, such as susceptibility to oxidative degradation, limited shelf life and an undesirable flavour, which can significantly affect their use in food products. These problems highlight the need for advanced processing techniques, such as encapsulation, to maintain the integrity and functional properties of fish protein hydrolysates (*7*).

In recent years, the encapsulation of protein hydrolysates has been a subject of extensive research, where various methods have been used to optimize this process. Spray-drying has proven to be the most common technique for encapsulating protein hydrolysates due to its ability to rapidly and efficiently convert liquid hydrolysate mixtures into stable powdered forms (*8*). Recent studies on protein hydrolysate encapsulation have mainly focused on the effects of carrier type, mass per volume ratio, and temperature using descriptive methods (*7*, *9*–*11*). However, there are few studies on optimizing encapsulation conditions at lower temperatures to improve product quality and preserve protein hydrolysate. These approaches are crucial as a more precise and scientifically sound understanding of the encapsulation parameters can improve the quality of the encapsulated product while mitigating the risks of thermal degradation and ensuring the preservation of the sensitive protein hydrolysate.

The aim of this study is to optimize the encapsulation of lemuru fish protein hydrolysate by spray-drying at a maximum temperature of 100 °C. Spray-drying at low temperatures (90–100 °C) is a milder process that protects sensitive substances such as enzymes, probiotics and proteins. The temperature range of 90–100 °C was chosen to maintain protein stability. We hypothesise that lower temperatures could improve the conversion rate of volatile fluids into powders by limiting evaporation. A higher temperature will break down the protein and reduce its functional properties (*12*, *13*). In addition, this technique provides numerous operational advantages, such as improved thermal efficiency, remarkable energy savings, reduced risk of corrosion and shorter processing time, all of which improve the effectiveness and sustainability of the drying process.

Using response surface methodology (RSM) and Box-Behnken design (BBD), we evaluated the effects of carrier type, mass per volume ratio and inlet temperature on the encapsulation efficiency of gum Arabic and maltodextrin, which were selected as liquid feeds due to their low viscosity and high solubility in water (*14*). The aim of this study is to determine the optimal encapsulation conditions that maintain the functional integrity of fish protein hydrolysate, contribute to the development of functional foods and introduce a new strategy for the encapsulation of fish protein hydrolysate.

## MATERIALS AND METHODS

### Materials

Fresh lemuru fish were purchased from a local market in Bandung City, Indonesia. Chemicals used in the encapsulation process included Alcalase enzyme (Xian Arisun ChemParm Co Ltd, Shaanxi, PR China), maltodextrin (Garuda Mas Lestari, Bandung, Indonesia), with a dextrose equivalent (DE) of 10–12, and gum Arabic (PT Brataco, Bekasi, Indonesia).

### Preparation of lemuru fish protein hydrolysate

Lemuru fish protein hydrolysate was produced according to the method of Priatni *et al.* (*10*). Frozen lemuru were thawed, mixed with distilled water in a ratio of 1:4, and homogenised in a blender. The pH of the mixture was adjusted to 6.0 using 0.1 M HCl solution. The hydrolysis was carried out in a a 15-litre bioreactor unit (Bangun Rahmat Teknik, Bandung, Indonesia) by adding 0.75 % (*m/V*) of Alcalase enzyme to the fish slurry and stirred in a reactor at 50 °C for 6 h. The process was stopped by heating at 85 °C for 15 min. Finally, the fish protein hydrolysate was filtered using a vacuum filter and stored at 20 °C.

### Encapsulation of lemuru fish protein hydrolysate using spray-drying method

Lemuru fish protein hydrolysate was encapsulated according to Kurozawa *et al.* (*15*). Carrier agents (maltodextrin, gum Arabic and maltodextrin) were added at mass per volume ratio of 10, 20 and 30 % directly to the lemuru fish protein hydrolysate and mixed under stirring until dissolving completely. The encapsulation was carried out using a spray dryer unit (Mitra Sentosa, Malang, Indonesia), designed specifically for fish protein hydrolysate processing. The mixture (1 L) was fed into the drying chamber for 2–3 h and the dried product was finally collected for analysis. The inlet temperature of spray dryer was 90, 95 or 100 °C and the outlet temperature was 80 °C with a feed flow rate of about 200 mL/h. The capacity of spray dryer was 1 L/h.

### Optimization of lemuru fish protein hydrolysate using response surface-Box-Behnken design

Response surface method and Box-Behnken design (RSM-BBD) were used to determine the optimum encapsulation conditions for lemuru fish protein hydrolysate using the spray-drying method. This statistical approach facilitated the modelling and analysis of three critical process variables: the carrier type, the carrier mass per volume ratio, and the spray-drying inlet temperature. RSM-BBD was designed for several experiments for the optimization process. The experiment was conducted in 15 repetitions. The optimal process was determined by evaluating several desirable responses, including high solubility, high yield and low hygroscopicity. The experimental data on yield, hygroscopicity and solubility were subjected to a comprehensive multivariate regression analysis using Minitab software v. 19.0 (*16*). This analysis describes the quantitative relationship between the process variables and each response. The multivariate regression model is:


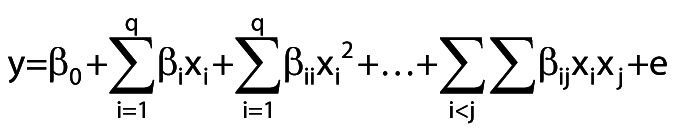
 /1/

where y is the response, β_0_ is offset term; β_i_, β_ii_ and β_ij_ are the regression coefficients; and x_i_ and x_j_ are the levels of the independent variables.

We conducted a validation experiment to empirically verify the predicted optimal parameters after determining the optimal conditions. This involved comparing the experimental results under these conditions against the predictions generated by Minitab software v. 19.0 (*16*), ensuring that the identified parameters lead to the desired encapsulation performance.

### Yield analysis

According to Agatha *et al.* (*17*), the spray-dried encapsulated lemuru fish protein hydrolysate was collected and weighed using an analytical scale. Yield (*Y*) percentage (*m*/*m*) of encapsulated lemuru fish protein hydrolysate was calculated using the following equation:


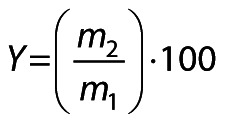
 /2/

where *m*_1_ is the mass of the lemuru fish protein hydrolysate in liquid form and carrier agent as fed in the spray dryer and *m*_2_ is the mass of the dried encapsulated lemuru fish protein hydrolysate.

### Hygroscopicity analysis

Hygroscopicity of the encapsulated sample was determined according to the method of Sarabandi *et al.* (*18*) with some modifications. A mass of 2 g of the samples was weighed, then placed in a Petri dish and stored in a desiccator containing saturated NaCl solution under a relative humidity of 76 % at room temperature (25 °C). After seven days, the samples were weighed on an analytical balance. The hygroscopicity of the lemuru fish protein hydrolysate was calculated using the following equation:


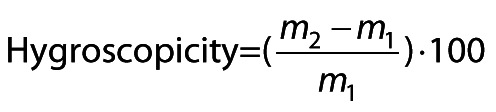
 /3/

where *m*_1_ is the mass of lemuru fish protein hydrolysate on the first day (2 g) and *m*_2_ is the mass of the final product after being stored for seven days.

### Water solubility

Water solubility was determined according to Sarabandi *et al.* (*18*), with some modifications. The sample powder (1 g) was dissolved in 100 mL of distilled water using a magnetic stirrer at 235×*g* for 4 min. The mixture was centrifuged (centrifuge model MX-301; TOMY, Tokyo, Japan) at 3000×*g* for 4 min. The supernatant (25 mL) was dried in an oven at 105 °C for 3 to 5 h and weighed on an analytical balance to determine the dry mass of the insoluble protein. The solubility percentage (*m*/*m*) was calculated using the following equation:


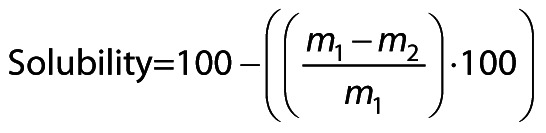
 /4/

where *m*_1_ is the initial mass of lemuru fish protein hydrolysate before drying (1 g) and *m*_2_ is the mass of the final product after drying.

### Analysis of functional groups by Fourier transform infrared spectroscopy

Functional groups present in both encapsulated and non-encapsulated lemuru fish protein hydrolysate were characterized using Fourier transform infrared (FTIR) spectroscopy (Nicolet iS5 iD5 ATR; Thermo Scientific, Waltham, MA, USA).This method was adapted and modified from the procedure outlined by Priatni *et al.* (*19*). The samples were dispersed homogeneously in potassium bromide to form discs. Spectral data were collected over a wavelength range from 4000 to 400 cm^-1^ at room temperature. The functional group of the encapsulated fish protein hydrolysate was compared to the non-encapsulated fish protein hydrolysate.

### Morphological surface of the fish protein hydrolysate particle determined by scanning electron microscopy

Particle morphology of the encapsulated and non-encapsulated fish protein hydrolysate was evaluated by scanning electron microscopy (SEM) (JSM-IT30; Jeol Ltd., Akhishima, Tokyo, Japan). Samples were put on the sample holder using a double conductive tape. The powders were coated with gold under vacuum and then examined by SEM.

### Particle size distribution analysis

Particle size of both encapsulated and non-encapsulated fish protein hydrolysate was evaluated according to Priatni *et al.* (*19*) with some modifications using particle size analyzer (PSA) (Zetasizer Nano ZS Malvern Panalytical, Malvern, UK). A mass of 1 g of sample was mixed with 10 mL of distilled water, centrifuged (MX-301; TOMY) at 3075×*g* for 30 min and examined with PSA. Particle size distribution was determined by the percentage (%) of particle diameter (nm).

### Statistical analysis

The data obtained were analyzed using analysis of variance (ANOVA) at 5 % level. All data analyses were done using Minitab v. 19.0 (*16*).

## RESULTS AND DISCUSSION

### Analysis of response data (yield, hygroscopicity and solubility)

In our study, the yield of lemuru fish protein hydrolysate encapsulated by spray-drying in a temperature range of 90 to 100 °C varied from 0.49 to 3.26 % (*m*/*m*). At an inlet temperature of 90 °C, combining gum Arabic and maltodextrin as carriers resulted in yields of less than 1.6 %. This observation is in contrast to the results of Hau *et al.* (*20*), who reported yields of 0.6 to 1.6 % (*m/V*) for protein hydrolysate from yellowstripe scad fish at an inlet temperature of 80 °C using an ultrasonic spray dryer. The lower yields at 90 °C indicate insufficient energy for optimal water evaporation, which is a significant factor for higher yields in spray drying. At this temperature, the evaporation rate slows down, increasing the moisture content of the final product and the possibility of water molecules to remain inside the drying chamber, thus increasing product loss (*21*). Conversely, higher inlet temperatures generate more thermal energy and destabilise water molecules. This increased energy facilitates the breaking of hydrogen bonds between water and active groups in the hydrolysate and enables more efficient and complete water evaporation (*21*).

**Fig. 1:**
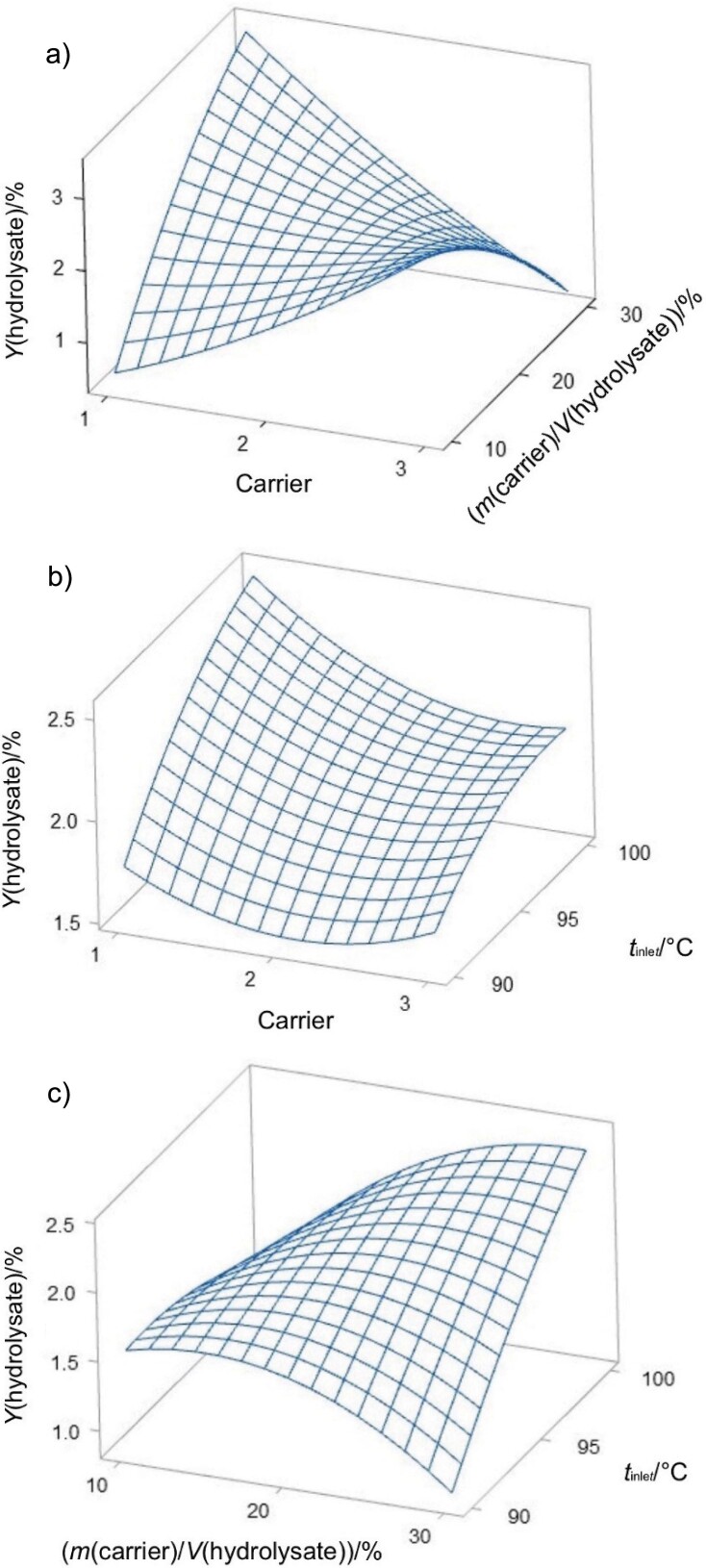
Surface plots of yield responses as a function of: a) type of carrier and *m*(carrier)/*V*(hydrolysate), b) type of carrier and inlet temperature, and c) *m*(carrier)/*V*(hydrolysate) and inlet temperature. Carrier: 1=maltodextrin, 2=maltodextrin and gum Arabic, and 3=gum Arabic

Response surface methodology (RSM) was used to further elucidate the relationship between the yield and the key variables of carrier type, mass per volume ratio and inlet temperature (Fig. 1). The yield surface plot in Fig. 1a indicates a negative correlation between gum Arabic mass per volume ratio and the yield. This negative correlation is due to the high sugar content of gum Arabic, which leads to stickiness and adhesion of the product to the chamber walls at higher temperatures, thus reducing the yield. The combination of maltodextrin and gum Arabic increases the yield compared to the use of gum Arabic alone, as mentioned by Yarlina *et al.* (*22*). An increase in the mass per volume ratio of maltodextrin shows an increase in the yield. This result is similar to observations from a study on spray-dried powder from red dragon fruit and kombucha, where a similar trend was observed (*17*). Fig. 1b shows an increase in yield to above 2.4 % (*m*/*m*) when the inlet temperature was increased from 90 to 100 °C, which is due to improved moisture evaporation and a resulting drier product (*23*). However, Fig. 1c shows an opposite scenario, where an increase in inlet temperature combined with higher carrier mass per volume ratio led to an observable decrease in yield from 2.2 to 1 % (*m*/*m*). This emphasises the complex mechanism of the encapsulation process and the critical balance required among temperature, carrier mass per volume ratio and encapsulation efficiency.

The hygroscopic nature of food products is an intrinsic property that significantly influences their quality, shelf-life and storage conditions. Hygroscopicity determines how much moisture a product can absorb from the environment, which is a crucial factor for the stability and longevity of the product (*24*). The hygroscopicity of the encapsulated lemuru fish protein hydrolysate ranged from 4.53 to 20.16 % mass fraction. This variation can be attributed to the inherent hydrophilic properties of both encapsulating agents used, maltodextrin and gum Arabic.

**Fig. 2:**
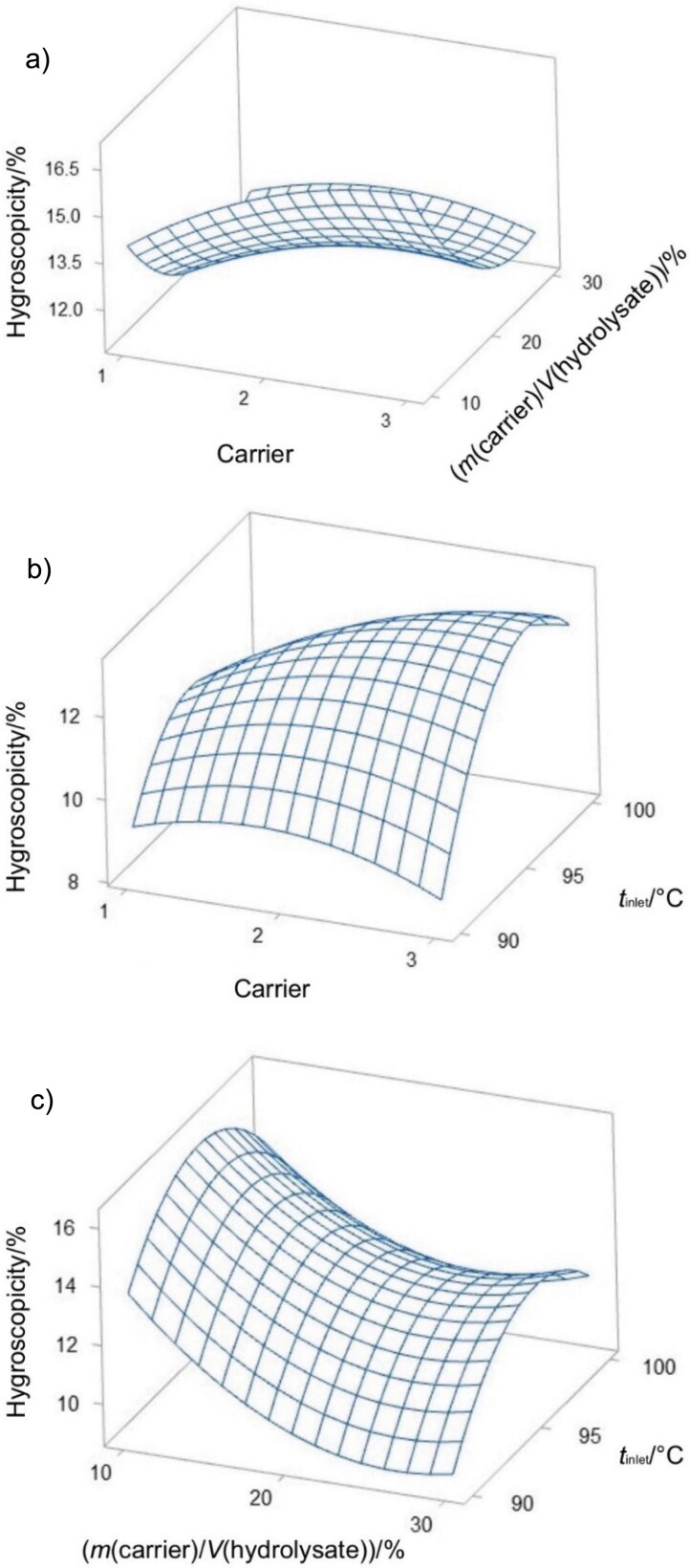
Surface plots of hygroscopicity as a function of: a) type of carrier and *m*(carrier)/*V*(hydrolysate), b) type of carrier and inlet temperature, and c) *m*(carrier)/*V*(hydrolysate) and inlet temperature. Carrier: 1=maltodextrin, 2=maltodextrin and gum Arabic, and 3=gum Arabic

The response surface plots in Fig. 2a show that a higher mass per volume ratio of gum Arabic reduces the hygroscopicity of the encapsulated lemuru fish protein hydrolysate. Higher amounts of gum Arabic effectively function as a barrier to moisture absorption. It forms a protective layer around the particles, preventing their exposure to moisture in the environmental (*25*). The combination of maltodextrin and gum Arabic reduces the hygroscopicity of the encapsulated lemuru fish protein hydrolysate. These results could be explained by the ability of maltodextrin to reduce hygroscopicity (*26*). This is in agreement with the findings of Sukri *et al*. (*27*), who suggest that the higher amount of hydrophilic groups in gum Arabic than in maltodextrin allows a better interaction with water, leading to increased moisture absorption.

Fig. 2b shows a direct correlation between inlet temperature and hygroscopicity, with an increase from 4.5 to 16 % (*m*/*m*) when the temperature rises from 90 to 96 °C. This relationship is probably due to a lower water vapour pressure gradient between the powder and the ambient atmosphere, resulting in better equilibrium moisture content (*28*). However, a decrease in hygroscopicity was observed at temperatures above 96 °C, a result that mirrors the results of Reshan Jayawardena *et al.* (*29*), who reported lower hygroscopicity of spray-dried beef lung powder at higher drying temperatures. This reduction may be due to protein denaturation and increased surface hydrophobicity, which can repel water molecules (*29*).

Fig. 2c shows that as carrier mass per volume ratio increases, the hygroscopicity of the encapsulated lemuru fish protein hydrolysate tends to decrease. The lower water uptake results from the ability of the carrier to interact with the hydrophilic groups of the protein hydrolysate. Furthermore, at an inlet temperature of 95 °C, the frequency of protein denaturation increases, which probably contributes to this reduced hygroscopicity. These results support the theory that the protein structure changes significantly at higher temperatures (*30*).

The solubility of fish protein hydrolysate in water is a key factor for its functional performance in food applications. High solubility is essential for the effective incorporation of fish protein hydrolysate into different food matrices, where it serves as an integral component in the formation of emulsions, foams and gels, and contributes to the texture and stability of the final product (*31*). The solubility of encapsulated lemuru fish protein hydrolysate ranges from 69.40 to 79.69 % (*m*/*m*), highlighting its suitability for such applications. The encapsulation process using maltodextrin as the carrier was found to significantly increase solubility compared to gum Arabic. This could be due to the lower molecular mass and higher dextrose equivalent of maltodextrin, which improves the interaction between the protein and water, and reduces intermolecular bonding, resulting in a better dispersed and soluble end product.

**Fig. 3:**
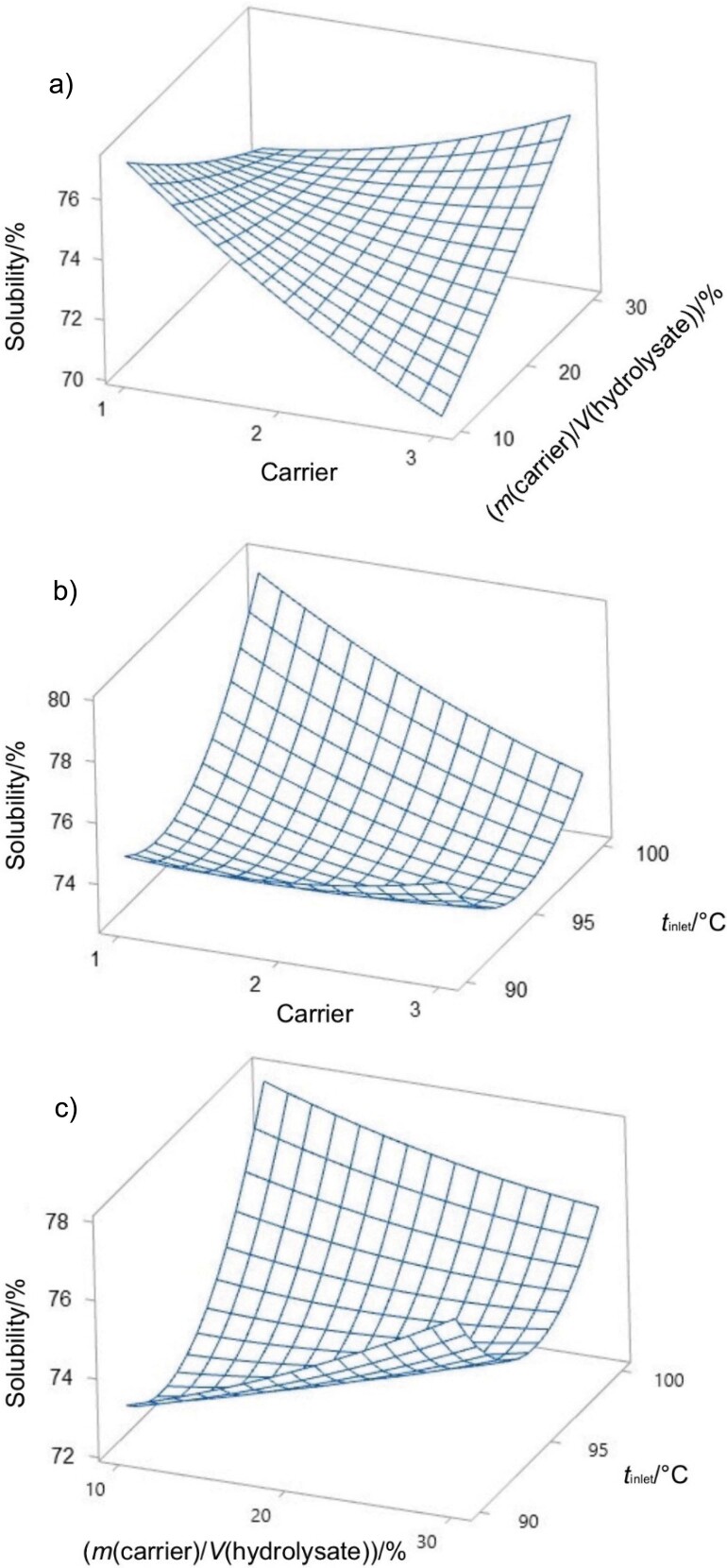
Surface plots of solubility as a function of: a) type of carrier and *m*(carrier)/*V*(hydrolysate), b) type of carrier and inlet temperature, and c) *m*(carrier)/*V*(hydrolysate) and inlet temperature. Carrier: 1=maltodextrin, 2=maltodextrin and gum Arabic, and 3=gum Arabic

Fig. 3a illustrates a decrease in the solubility of encapsulated lemuru fish protein hydrolysate with increasing mass per volume ratio of maltodextrin. This trend suggests that maltodextrin may interact with the hydrophilic sites of the protein, potentially forming a semi-impermeable matrix that inhibits the interaction of the protein with water molecules. As the content of maltodextrin increases, the barrier effect intensifies, resulting in lower solubility in aqueous environment, which is consistent with the observations of Ningsih *et al.* (*32*). Conversely, encapsulation with gum Arabic, which is rich in dietary fibre, appears to improve the dissolution of lemuru fish protein hydrolysate, possibly due to the ability of hydrocolloids to improve the water retention and solubility of the protein.

It is important to recognize that both maltodextrin and gum Arabic play essential roles in preserving the structural integrity of the protein during drying. Their protective effect is crucial for maintaining the functional solubility properties of the protein, as mentioned by Siregar *et al.* (*33*). Fig. 3b shows that solubility exceeds 79 % (*m*/*m*) as the drying inlet temperature increases. This increase in solubility can be attributed to the lower hygroscopic property of the powder at higher temperatures. It has a positive effect on solubility and is in agreement with findings from Araújo *et al.* (*34*). However, lemuru fish protein hydrolysate encapsulated in gum Arabic shows a significant decrease in solubility when processed at temperatures ranging from 90 to 96 °C. This decrease in solubility could be caused by particle agglomeration, which makes particle reconstitution in water more difficult. Fig. 3c emphasises this point by showing how agglomeration at lower temperatures reduces solubility. Above 96 °C, the hydrogen bonds between the hydrophilic groups and the water molecules are likely to be disrupted, leading to increased water evaporation and a decrease in protein solubility.

### Results of RSM-Box-Behnken design

The encapsulation of fish protein hydrolysate can be effectively modelled using the response surface methodology (RSM) with Box-Behnken design, as shown by the regression equation expressed as a second-order polynomial equation. Table S1 shows the input experimental data of the responses for RSM optimization.

As shown in Table S2, this equation contains constant terms, linear and quadratic coefficients, and cross-product interaction terms to account for the interdependent effects of carrier type (x1), carrier content (x2) and inlet temperature (x3). Table 1 shows the non-significant lack-of-fit values for the yield and solubility responses, with the regression model close to 1 and p>0.05. This confirms that the predictions of the model are consistent with the experimental data and supports the statistical robustness of the model. The model illustrates a significant interaction (p≤0.05) between the type of carrier and its content, particularly for the yield response. This indicates that these factors work together to influence the yield of the encapsulated fish protein hydrolysate and that maximising the encapsulation process depends critically on how these variables interact.

**Table 1:** The p-values of each response

Term	Yield/%	Hygroscopicity/%	Solubility/%
	p-value		
Model	0.182	0.791	0.206
Carrier agent (x_1_)	0.557	0.590	0.204
Carrier mass per volume ratio (x_2_)	0.609	0.254	0.567
Inlet temperature (x_3_)	0.281	0.663	0.220
x_1_ x_1_	0.617	0.731	0.684
x_2_ x_2_	0.402	0.445	0.811
x_3_ x_3_	0.745	0.300	0.075
x_1_ x_2_	0.011*	0.723	0.060
x_1_ x_3_	0.762	0.603	0.209
x_2_ x_3_	0.227	0.844	0.226
Lack-of-fit	0.093**	0.050	0.781**
Regression model	0.80	0.50	0.79

*Significant value (p≤0.05), **close to 1 (p>0.05)

**Fig. S1:**
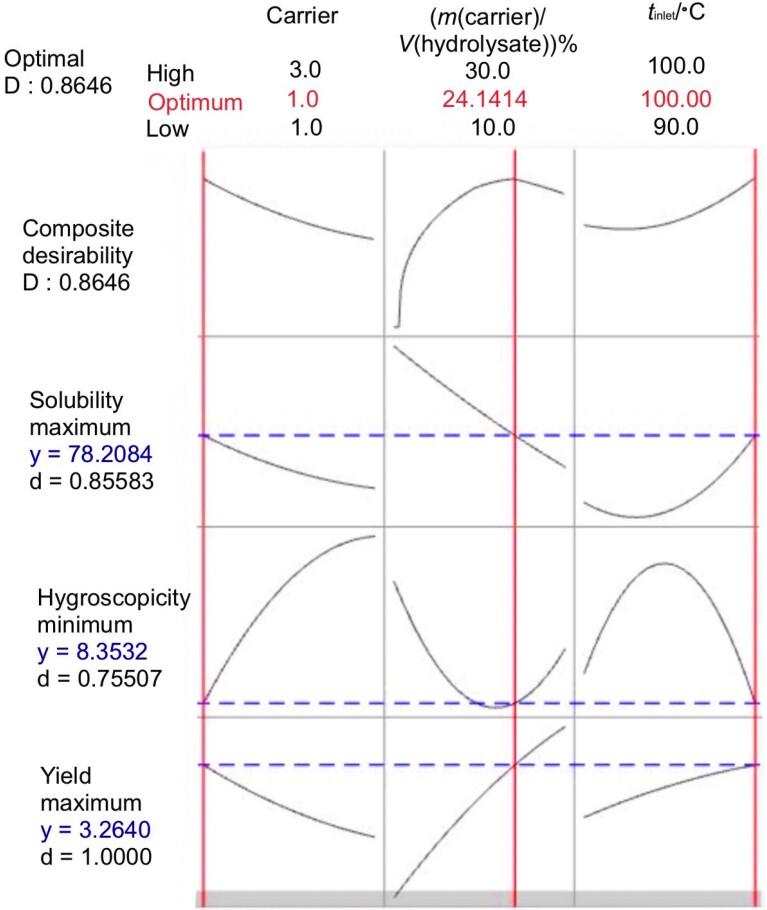
The desirability of the responses. D=desirability, Carrier 1.0=maltodextrin

Based on the RSM-Box-Behnken analysis, the optimal conditions for the encapsulation of fish protein hydrolysate were determined when using 25 % (*m/v*) maltodextrin as the carrier and an inlet temperature of 100 °C. These parameters achieved a high desirability index of 0.864, suggesting an effective balance among yield, solubility and hygroscopicity (Fig S1). In addition, all the responses of the products were validated by laboratory experiments. The actual and predicted values can be found in Table S3. The data showed that the actual and predicted values were within the range (95 % prediction interval); thus, the reliability of the optimized conditions was verified. The predicted response values were obtained by calculating the experimental data in Table S1 using the equations in Table S2. The results were validated by comparing actual and predicted values of each response.

Due to their favourable physicochemical properties, maltodextrins are often chosen as encapsulating matrices in spray-drying applications. They have high solubility and low hygroscopicity, which is crucial for the stability of the encapsulated products. Additionally, maltodextrins maintain low viscosity at high amounts, facilitating efficient spray-drying processes (*35*). Kurozawa *et al.* (*15*) highlighted the superiority of maltodextrin as an encapsulating agent for chicken protein hydrolysate in spray-drying, pointing out its ability to yield products with lower hygroscopicity than gum Arabic. This is particularly favourable for the storage and handling of dried products. Furthermore, various studies suggest that the optimal mass per volume ratio of maltodextrin as an encapsulating material is between 25 and 30 %. Within this mass per volume ratio threshold, maltodextrin offers optimal powder recovery and encapsulation efficiency, as evidenced by the high-quality properties of the encapsulated material (*36*). The ability of maltodextrin to form an amorphous and porous matrix contributes to its effectiveness in encapsulation, supporting the entrapment of volatile compounds and the protection of sensitive ingredients from oxidative damage.

Using the robust RSM-Box-Behnken design optimization framework, a sample of encapsulated lemuru fish protein hydrolysate was selected and produced under the most favourable conditions. These conditions were determined to be a maltodextrin mass per volume ratio of 25 % and a spray-drying inlet temperature of 100 °C. We assume that the use of a lower temperature of 100 °C for spray-drying fish protein hydrolysate is favourable for several reasons. In our study, a spray dryer was designed for inlet temperatures between 90 and 100 °C. This ensures the preservation of sensitive bioactive compounds that could be otherwise degraded at higher temperatures, thus maintaining the nutritional and functional integrity of the fish protein hydrolysate. This temperature optimizes the efficiency of moisture removal without the excessive energy costs associated with higher temperatures, making the process more economical and environmentally sustainable.

### Functional groups

**Fig. 4:**
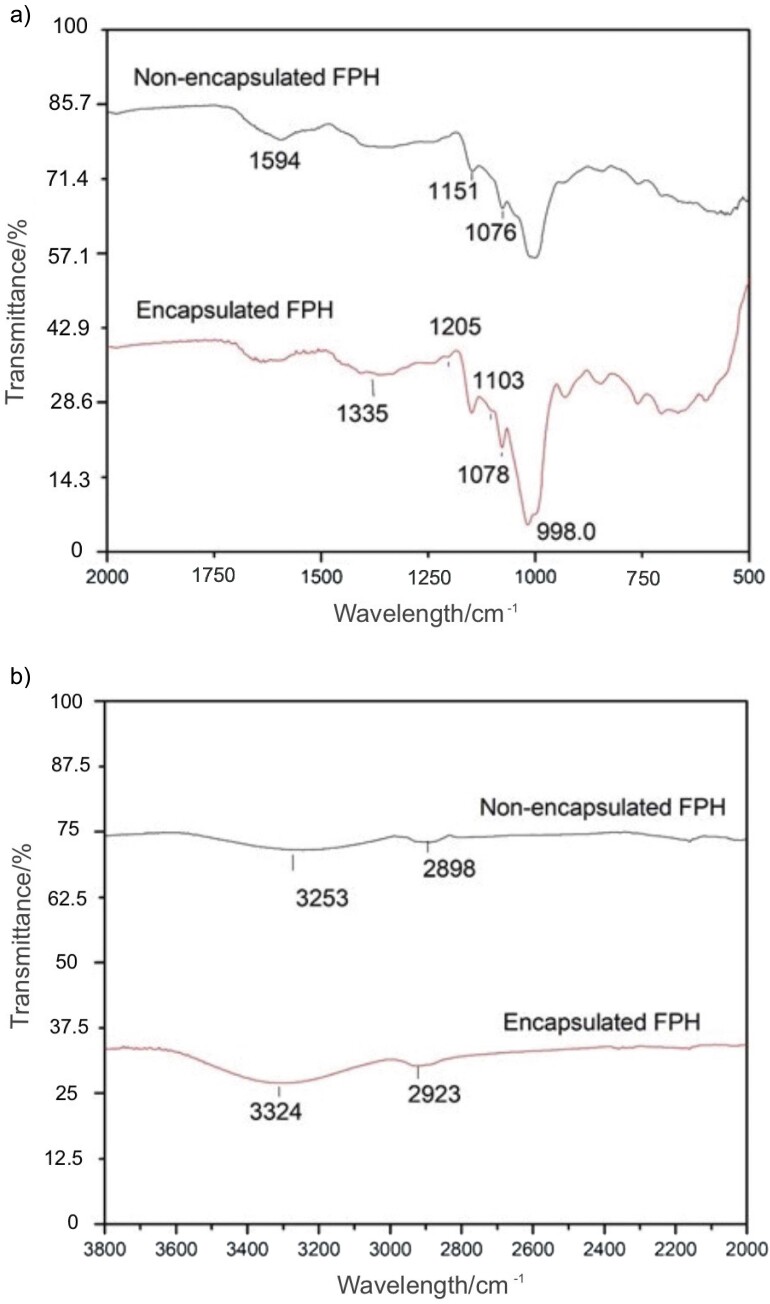
FTIR spectra of non-encapsulated and encapsulated lemuru fish protein hydrolysate (FPH) in the range of wavelengths: a) 500 to 2000 cm^-1^ and b) 2000 to 3000 cm^-1^

Fig. 4 shows the Fourier transform infrared (FTIR) spectra of lemuru fish protein hydrolysate in encapsulated and non-encapsulated forms. The spectra show characteristic absorption bands for different functional groups. For both sample types, the NH_3_ deformation in free amino acids such as lysine is indicated in the spectral region of 1050-1000 cm^-1^. The encapsulated fish protein hydrolysate shows a C-N stretch at 1078 cm^-1^, slightly shifted from the 1076.75 cm^-1^ peak of the non-encapsulated fish protein hydrolysate, suggesting a possible interaction of the carrier matrix with the amino groups. Amide I bands, indicative of protein secondary structure, appear at 1631 cm^-1^ for the encapsulated fish protein hydrolysate and at 1594 cm^-1^ for the non-encapsulated fish protein hydrolysate, which may indicate some conformational changes due to the encapsulation process. The peaks at 1335 and 1366 cm^-1^ are attributed to O-H bending vibrations in alcohol functional groups, a sign of polysaccharides such as maltodextrin in the encapsulated fish protein hydrolysate. Furthermore, the broader O-H stretching vibration observed at 3324 cm^-1^ for encapsulated fish protein hydrolysate compared to 3253 cm^-1^ for non-encapsulated fish protein hydrolysate, together with a shift in the C-H stretching vibrations from 2898 to 2923 cm^-1^, indicates an increased number of hydroxyl groups due to encapsulation. These O-H and N-H functional groups are integral to the water-binding capacity of fish protein hydrolysate, and the observed shifts suggest that encapsulation may affect the moisture interaction and solubility of the hydrolysate.

The FTIR spectral analysis between the wavelengths 3750 and 1000 cm^-1^ shows a decrease in transmittance for the encapsulated lemuru fish protein hydrolysate compared to its non-encapsulated counterpart. This decrease in transmittance indicates a denser molecular packing within the encapsulated product, likely due to the formation of a molecular complex between the maltodextrin polysaccharide carrier and the core protein molecules (*37*). A comparison of encapsulated and non-encapsulated lemuru fish protein hydrolysate shows clear differences in the functional groups. The encapsulated form shows a C-O stretching band at 1205 cm^-1^ that is characteristic of the glycosidic bonds in glucose unit of maltodextrin, which is consistent with Maqsoudlo *et al.* (*38*). According to Castro-Cabado (*39*), the peaks at 998 and 1103 cm^-1^ in the encapsulated fish protein hydrolysate spectra can be interpreted as the stretching vibrations of anhydroglucose rings, suggesting the formation of these structures as a result of the encapsulation process. This formation occurs through the glycosidic binding of glucose units from maltodextrin with protein molecules, coupled with the release of water molecules. The O-H stretching vibration in the encapsulated fish protein hydrolysate shifts to 3324 cm^-1^, indicating potential hydrogen bonding interactions between the fish protein hydrolysate and maltodextrin. The sharper and more defined peak at this wavenumber for the encapsulated fish protein hydrolysate than the broader peak at 3253 cm^-1^ for the non-encapsulated version indicates a modified hydrogen bonding environment. This sharpening of the O-H peak indicates a structured environment around the hydroxyl groups, which could correlate with improved water solubility of the encapsulated product.

### Particle morphology

**Fig. 5:**
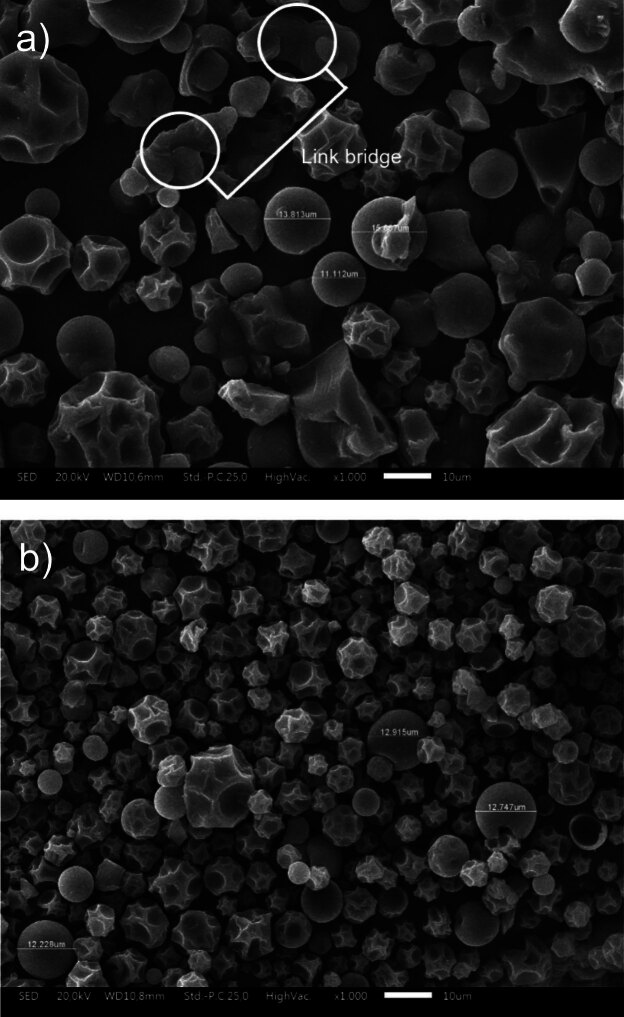
Scanning electron microscope photographs of: a) non-encapsulated and b) encapsulated lemuru fish protein hydrolysate with 1000× magnification

Fig. 5 shows SEM images that contrast the microstructure of non-encapsulated and optimally encapsulated lemuru fish protein hydrolysate. Quantitative image analysis shows that the average particle size of the non-encapsulated fish protein hydrolysate is approx. 13.53 µm, while the encapsulated fish protein hydrolysate has a slightly smaller average size of 12.2 µm. This size reduction of the encapsulated particles can be attributed to the densification effect during the encapsulation process. Moreover, the encapsulated particles have a characteristically wrinkled surface topology, a consequence of the rapid moisture evaporation enabled by spray-drying at high temperature and the formation of a carbohydrate matrix from maltodextrin. This surface morphology is indicative of the film formation and drying kinetics that are unique to the encapsulation process.

The SEM image shows that the non-encapsulated fish protein hydrolysate particles appear to have a smoother surface texture than the encapsulated fish protein hydrolysate. This effect occurs because the thermal expansion of the gum Arabic trapped within the droplets exerts pressure on the formation of particle walls, resulting in a smooth and taut surface. Fig. 5a shows the presence of link bridges between particles of non-encapsulated fish protein hydrolysate, which is probably due to the higher hygroscopicity of the material. These link bridges, as observed in the results of Kurozawa *et al.* (*15*), are attributed to the tendency of non-encapsulated protein hydrolysates to absorb moisture, leading to particle agglomeration. It is hypothesised that the encapsulation process, particularly with maltodextrin, reduces these hygroscopic interactions thus reducing the tendency to form link bridges and possibly improving the flowability and stability of the powder.

### Particle size distribution

**Fig. 6:**
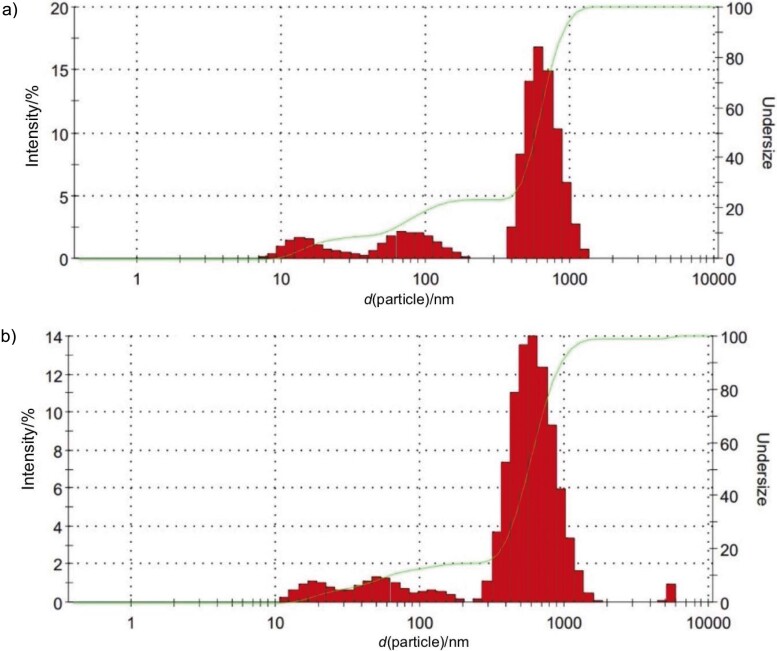
Particle size analyser result of: a) encapsulated (*d*(particle)_Z-average_=520.2 nm) and b) non-encapsulated (*d*(particle)_Z-average_=428.8 nm) lemuru fish protein hydrolysate

Particle size analysis, as shown in Fig. 6, shows a narrow particle distribution in the lemuru fish protein hydrolysate encapsulated with a maltodextrin mass per volume ratio of 25 %, with sizes ranging from 396.1 to 1281 nm and a mean diameter of 520.2 nm, indicating a Z-average value. In contrast, the non-encapsulated fish protein hydrolysate samples had a broad particle distribution, from 255.0 to 1718 nm, and a smaller mean particle diameter of 428.8 nm, as indicated by their respective Z-average. The encapsulated samples show a more uniform particle size distribution than their non-encapsulated counterparts. This uniformity can be attributed to the viscosity of the mixture of fish protein hydrolysate and maltodextrin, which forms a more cohesive matrix during-spray drying. The higher viscosity helps to stabilise the droplets as they pass through the nozzle, resulting in reduced expansion and more uniform particle size despite the forces of thermal expansion (*38*). The narrow distribution of encapsulated particles improves the solubility of the encapsulated lemuru fish protein hydrolysate. A larger surface area allows a better interaction with the solvent, which can increase the rate and extent of dissolution.

## CONCLUSIONS

In conclusion, in this study the encapsulation of lemuru fish protein hydrolysate was effectively optimized by spray-drying techniques. Using the RSM-Box-Behnken design, it was found that the mass per volume ratio of maltodextrin of 25 % and an inlet temperature of 100 °C are the ideal conditions to achieve the best quality of the encapsulated product. Under these conditions, a significant interaction (p≤0.05) was observed between the carrier type and mass per volume ratio affecting the yield of encapsulated fish protein hydrolysate. Moreover, the model predictions for yield and solubility were validated, as indicated by a non-significant lack-of-fit values (p>0.05). Encapsulated lemuru fish protein hydrolysate was shown to have lower hygroscopicity than its non-encapsulated counterpart, as evidenced by the reduced formation of link bridges and highlighted in SEM imaging. Further analytical evaluations using FTIR and PSA suggest that encapsulation increases the solubility of the lemuru fish protein hydrolysate, possibly due to the increased presence of functional O-H groups and a larger specific surface area of the maltodextrin carrier. These results emphasise the value of encapsulation in improving the functional properties of fish protein hydrolysate and provide an understanding of the physical and chemical properties of the food application.

## Appendix

**Table S1:** Experimental data for the optimization using response surface methodology

Run	Carrier	(*m*(carrier)/*V*(hydrolysate))/%	*t*_inlet_/°C	*Y*(EFPH)/%	Hygroscopicity/%	Solubility/%
1	2	10	100	1.394	12.185	78.684
2	1	10	95	0.505	11.710	76.084
3	2	30	90	0.694	11.080	75.920
4	1	20	100	2.353	12.450	79.692
5	3	20	100	1.403	10.900	74.713
6	3	20	90	1.858	4.525	75.403
7	2	20	95	2.117	12.810	75.043
8	2	20	95	1.633	11.525	74.609
9	1	20	90	2.384	10.465	74.713
10	3	10	95	3.264	20.155	69.403
11	3	30	95	0.489	14.155	76.880
12	1	30	95	2.951	8.680	74.027
13	2	30	100	2.981	10.305	74.963
14	2	10	90	0.931	14.600	74.217
15	2	20	95	2.052	13.815	70.547

Carrier 1=maltodextrin, carrier 2=maltodextrin and gum Arabic, carrier 3=gum Arabic, EFPH=encapsulated fish protein hydrolysate

**Table S2:** Regression equation of each response

Response/%	Regression equation
Yield	
Hygroscopicity	
Solubility	

**Table S3:** Prediction and actual value of each response

Response/%	Prediction	Actual	PI_low_	PI_high_
Hygroscopicity	8.35	7.66	5.37	22.08
Solubility	78.21	76.20	71.39	85.03
Yield	3.28	3.07	0.9628	5.585

PI=prediction interval in the range of 95 %
